# Temporal Alignment of Predictors and Intended Clinical Use in AI‐Based Mortality Prediction for Cardiovascular Patients With COVID‐19

**DOI:** 10.1002/hsr2.72765

**Published:** 2026-07-01

**Authors:** Carlos M. Ardila

**Affiliations:** ^1^ Saveetha Dental College and Hospital, Saveetha Institute of Medical and Technical Sciences, SIMATS Saveetha University Chennai Tamil Nadu India; ^2^ Basic Sciences Department, Biomedical Stomatology Research Group, Faculty of Dentistry Universidad de Antioquia U de A Medellín Antioquia Colombia

## Conflicts of Interest

The author declares no conflicts of interest.

Dear Editor,

I read with great interest the recent article by Nopour on artificial intelligence models for predicting COVID‐19 mortality among patients with cardiovascular disease [1]. The study addresses a clinically important question, and the use of SHAP analysis adds welcome interpretive value to the reported model performance. In particular, the identification of age, pneumonia, ICU admission, type of surgery, and d‐dimer as influential variables provides a potentially useful framework for discussing risk stratification in this vulnerable population.

A central question, however, seems essential for interpreting the study's conclusions: at what exact clinical timepoint is the model intended to be used? This issue is especially important in prediction research because the intended moment of use, the prediction horizon, and the availability of predictors at that moment should be explicitly aligned if a model is to support real clinical decisions [2, 3]. In the present study, predictor values were assessed 2–3 days after COVID‐19 confirmation, whereas the conclusion states that the XGB model can identify at‐risk patients “on admission” [1].

This distinction is not merely semantic. Several of the most influential predictors highlighted by the SHAP analysis, especially ICU admission and type of surgery, may not function as baseline admission variables in the same way as age or an initial laboratory value. Rather, they may reflect evolving severity, treatment escalation, or features of the hospitalization that become apparent only after the initial presentation. More broadly, recent methodological guidance has emphasized that prediction models should be developed and evaluated according to their intended clinical use, since apparent performance can be difficult to interpret when predictors are not clearly anchored to the target decision point [2, 3, 4].

That point matters directly for the article's conclusions. If variables such as ICU admission or surgery‐related factors were not consistently available at the time the model is proposed to guide triage, then the model may be better understood as an early in‐hospital prognostic tool rather than a true admission model. Such a distinction affects not only clinical interpretation, but also the way readers judge transportability, workflow integration, and the plausibility of real‐world implementation [3, 4].

For this reason, it would be helpful if the author could clarify the intended prediction horizon and the temporal anchoring of the principal predictors, particularly ICU admission and surgery‐related variables. A clearer separation between baseline admission data and post‐admission evolving variables would strengthen the interpretation of the study and better define its clinical use case. This is a relevant issue beyond the present article, because in AI‐based prognostic research, usefulness depends not only on discrimination metrics but also on whether model inputs genuinely match the moment at which clinicians are expected to act [2, 3].

## Author Contributions

**Carlos M. Ardila:** conceptualization, investigation, funding acquisition, writing – original draft, methodology, validation, visualization, writing – review and editing, formal analysis, supervision.

## Ethics Statement

The author has nothing to report.

## Data Availability

The author has nothing to report.
